# Differential effect on myelination through abolition of activity‐dependent synaptic vesicle release or reduction of overall electrical activity of selected cortical projections in the mouse

**DOI:** 10.1111/joa.12974

**Published:** 2019-03-22

**Authors:** Kim V. Korrell, Jolande Disser, Kristina Parley, Auguste Vadisiute, Maï‐Carmen Requena‐Komuro, Harriet Fodder, Charlotte Pollart, Graham Knott, Zoltán Molnár, Anna Hoerder‐Suabedissen

**Affiliations:** ^1^ Department of Physiology, Anatomy and Genetics University of Oxford Oxford UK; ^2^ Swammerdam Institute for Life Sciences University of Amsterdam Amsterdam The Netherlands; ^3^ Institute of Molecular Biology and Medicine (IBMM) Université Libre de Bruxelles (ULB) Gosselies Belgium; ^4^ EPFL SV PTECH PTBIOEM AI 0143 (Bâtiment AI) Lausanne Switzerland; ^5^ Present address: VIB‐KU Leuven Center for Brain& Disease Research 3000 Leuven Belgium; ^6^ Present address: Department of Neurosciences Leuven Brain Institute, KU Leuven 3000 Leuven Belgium; ^7^ Present address: University College London, UK

**Keywords:** Kir2.1, layer VI and V projection neurons, myelination, Node of Ranvier, Ntsr1‐Cre, Rbp4‐Cre, SNAP25

## Abstract

Myelination of axons by oligodendrocytes in the central nervous system is crucial for fast, saltatory conduction of action potentials. As myelination is central for brain development and plasticity, and deficits are implicated in several neural disorders such as multiple sclerosis, major depressive disorder, bipolar disorder and schizophrenia, it is important to elucidate the underlying mechanisms regulating myelination. Numerous mechanisms have been proposed by which the communication between oligodendrocytes and active axons may regulate the onset and maintenance of activity‐dependent myelination. We compared two models of ‘silencing' layer V and/or VI cortical projection neurons from early stages by either decreasing their excitability through Kir2.1 expression, an inward rectifying potassium channel, introduced through *in utero* electroporation at embryonic day (E)13.5, or inhibiting regulated vesicular release through Cre‐dependent knock‐out of synaptosomal associated protein 25 kDA (SNAP25). SNAP25 is a component of the soluble N‐ethylmaleimide fusion protein attachment protein receptor (SNARE) complex, which, among others, is needed for calcium‐dependent regulated vesicle release from synapses. In layer VI cortical projection neurons in the *Ntsr1‐Cre;Ai14;Snap25* ^*fl/fl*^ mouse, we found that inhibiting regulated vesicular release significantly decreased the amount of myelin basic protein (MBP, used as marker for myelination) and the amount of myelinated projections at postnatal day (P)14 without affecting the initial timing of onset of myelination in the brain (at P7/P8). Additionally, overall oligodendrocyte maturation appears to be affected. A strong trend towards reduced node of Ranvier (NoR) length was also observed in *Ntsr1‐Cre;Ai14;Snap25* ^*fl/fl*^ corpus callosum. An equally strong trend towards reduced NoR length was observed in *Rbp4‐Cre;Ai14;Snap25* ^*fl/fl*^ corpus callosum at P14, and the g‐ratio in the spinal cord dorsal column was reduced at P18. However, no measurable differences in levels of MBP were detected in the striatum when comparing *Rbp4‐Cre;Ai14;Snap25* ^*fl/fl*^ and control brains. Conversely, Kir2.1 *in utero* electroporation at E13.5 did not significantly affect the amount of MBP or number of myelinated callosal axons at P14 but did significantly decrease the NoR length measured in the corpus callosum. It therefore seems likely that the excitability of the neuron can potentially perform a modulating function of myelin characteristics, whereas regulated vesicular release has the potential to have a more pronounced effect on overall myelination, but in a cell‐type specific manner.

## Introduction

During development of the central nervous system (CNS) of mammals and other vertebrates, oligodendrocytes extend membrane processes that wrap axons in a lipid‐rich myelin membrane. Myelination enables more rapid and less energy‐consuming action potential propagation by relatively thin axons, facilitating the evolution of a complex yet compact CNS in vertebrates (Fields, 2015). During rat CNS development, myelination progresses in a rostral to caudal gradient for the optic nerve and tract (Skoff et al. 1980), as well as the spinal cord (Schwab & Schnell, 1989). There are marked differences in the myelination of various CNS pathways. For example, many spinal cord tracts are fully myelinated before the onset of myelination in the major white matter tracts of the brain. Within the mouse corpus callosum, myelination is first detectable at postnatal day (P)11 and the percentage of myelinated axons increases rapidly for several months, but continues to change at least until P240 (Sturrock, 1980). Axons with larger diameter become preferentially myelinated (mean diameter 0.46 μm for myelinated and 0.25 μm for unmyelinated axons in the mouse corpus callosum). However, not all CNS axons become myelinated (Sturrock, 1980), and myelinated axons do not necessarily have uniform amounts of myelin along their entire length (Tomassy et al. 2014). Moreover, myelination can occur in the absence of any neural signals from the fibres. Oligodendrocytes even ensheathe synthetic nanofibres *in vitro*, provided the fibre diameter is > 0.4 μm (Lee et al. 2012). However, there is considerable overlap between the axon diameters of myelinated and unmyelinated axons, and little change in the distribution of axon diameters in the corpus callosum during the time of peak myelination in mice (Sturrock, 1980). Thus, although axon diameter is clearly one determinant of whether myelination occurs or not, it cannot be the only factor regulating which axons become myelinated *in vivo*.

There is increasing evidence that myelination is regulated by neuronal activity (Bechler et al. 2018). It has been suggested that myelination has two phases – intrinsic and then adaptive – during which axons become more myelinated (Bechler et al. 2018). Treatment of dissociated mouse brain cultures with the voltage‐gated sodium channel blocker tetrodotoxin (TTX) reduced the number of myelinated segments in these cultures (Demerens et al. 1996). Using an *in vitro* cell culture model of dorsal root ganglia neurons, Wake et al. (2011) showed that application of either tetanus toxin (TnTx) or botulinum toxin (BnTx), both of which prevent vesicular release, reduced the number of myelin segments per cell (Wake et al. 2011). In similar cell cultures with a mixture of neurons incapable of vesicle fusion and control neurons, the control axons were preferentially myelinated (Wake et al. 2015), demonstrating that vesicle fusion‐mediated communication between axons and oligodendrocytes plays a role in axon selection for myelination, at least *in vitro*.

In zebrafish, preventing synaptic vesicle exocytosis via expression of TnTx light chain resulted in reduced myelination along the axon (Hines et al. 2015; Mensch et al. 2015). When axons were wrapped, nascent sheaths were shorter in length (Hines et al. 2015) or the average number of sheaths from each oligodendrocyte was reduced (Mensch et al. 2015). Hines et al. (2015) demonstrated that treatment with TTX reduced the proportion of nascent myelin sheaths wrapping silenced axons, without affecting sheath length (Hines et al. 2015). Similarly, using Kir2.1 electroporation in zebrafish larvae to reduce axonal excitability, it was demonstrated that Kir2.1‐expressing axons were myelinated less frequently than controls, but sheath length was normal (Hines et al. 2015). Kir2.1 over‐expressing axons in the mouse corpus callosum were less frequently surrounded by MBP^+^ myelin rings (Mitew et al. 2018). Conversely, if axonal activity was increased using hM3Dq DREADD (designer receptor activated by designer drug) or optogenetic stimulation in mouse brains, the more active axons were preferentially ensheathed by MBP^+^ myelin in the corpus callosum (Mitew et al. 2018), more of their axonal length was covered by myelin (Stedehouder et al. 2018), or their g‐ratio decreased (Gibson et al. 2014).

All of the previous *in vitro* or *in vivo* studies implicating vesicle fusion in activity‐dependent myelination processes used acute expression of bacterial toxins to disrupt vesicle fusion. Synaptosomal‐associated protein 25 kDa (SNAP25) is an essential component of the SNARE (soluble N‐ethylmaleimide fusion protein attachment protein receptor) complex, and the protein targeted by TnTx. The SNARE complex is necessary for the regulation of vesicular release (Rizo & Südhof, 2012), including calcium‐dependent vesicle release from synapses. Ablation of SNAP25 abolishes the regulated vesicular release but does not affect spontaneous neuroexocytosis or non‐vesicular constitutive release (Washbourne et al. 2002). Ablation of SNAP25 from the entire nervous system gives insight into the activity‐dependent embryonic development *in vivo* (Molnár et al. 2002) or early postnatal events of axon growth and termination *in vitro* (Blakey et al. 2012). Despite normal embryonic CNS development, global SNAP25 KO leads to neonatal death due to respiratory failure. Therefore, in this publication, we take advantage of a recently described transgenic mouse suitable for cell‐type specific knock‐out of SNAP25 (Hoerder‐Suabedissen et al. 2018b). To study the effect of the regulated synaptic vesicular release on early myelination in two different white matter tracts, the corpus callosum or striatum, we used SNAP25 conditional‐knockout in two selected cortical projection neuron populations [Rbp4‐Cre layer V (LV) neurons and Ntsr1‐Cre layer VI (LVI) neurons; Grant et al. 2016]. We also examined the myelination in cortical projection neurons where we decreased their excitability through an inward rectifying potassium channel Kir2.1, expressed following *in utero* electroporation at E13.5. Electroporation at this time point targets a mixed LV and LVI cortical cell population, allowing some comparisons with the LV and LVI SNAP25 cKO. We investigated the time‐point of myelination onset using MBP immunohistochemistry, the progression of myelination using Olig2 and CC1 double immunohistochemistry, the amount of MBP present in the cortex or white matter tracts at P14, and the length of nodes of Ranvier (NoRs). Additionally, we report g‐ratio alterations in LV SNAP25 cKO spinal cord dorsal columns compared with controls.

## Methods

### Breeding and maintenance of transgenic mice

All animal experiments were approved by a local ethical review committee and conducted in accordance with personal and project licences under the UK Animals (Scientific Procedures) Act (1986).

B6‐Snap25tm3mcw (*Snap25* ^*fl/fl*^) mice were crossed with B6;129S6‐Gt(ROSA)26Sortm14(CAG‐tdTomato)Hze/J (*Ai14*) mice until both alleles were present homozygously. They were crossed with two Cre‐recombinase‐expressing strains (Tg(Rbp4‐cre)KL100Gsat/Mmucd (*Rbp4‐Cre;* Jackson Laboratories) and Tg(Ntsr1‐cre)GN220Gsat (*Ntsr1‐Cre;* Jackson Laboratories). Control mice were obtained by crossing *Cre/* ^*+*^ *;Ai14/Ai14* females with *Ai14/Ai14* males. Conditional knockout and heterozygous knockout mice were obtained by crossing *Cre/* ^*+*^ *;Snap25* ^*fl/+*^ *;Ai14* females (Rbp4‐Cre) or *Cre/* ^*+*^ *;Snap25* ^*fl/fl*^ *;Ai14* females (Ntsr1‐Cre) with *Snap25* ^*fl/fl*^;*Ai14/Ai14* males.

### *In utero* electroporation

Timed‐pregnant female C57BL\6 mice carrying E13.5 embryos were anaesthetised and subjected to abdominal incision to expose the uterus. Embryos were visualised through the uterine wall and the plasmids (2 mg μL^−1^) were injected unilaterally into the lateral ventricle followed by electroporation (five 50‐ms pulses at 40 V). The uterine horns were returned to the abdominal cavity, and the abdominal wall and skin were sutured or stapled, respectively (for our detailed protocols see Martinez‐Garay et al. 2016). Females were permitted to deliver normally, and brains of electroporated embryos (and unelectroporated littermates) were collected at P14. The plasmids used were pCAG‐Kir2.1‐T2A‐tdTomato (expressing Kir2.1 and tdTomato) and pCAG‐Kir2.1Mut‐T2A‐tdTomato (expressing a mutated non‐conducting Kir2.1 channel and tdTomato), which were a gift from Massimo Scanziani (Addgene plasmid # 60598 and # 60644, respectively (Xue et al. 2014)).

### Immunofluorescence to detect myelin‐related proteins

Brains for immunohistochemistry at various postnatal ages were perfusion‐fixed with 4% formaldehyde (F8775; Sigma‐Aldrich) in 0.1 m phosphate‐buffered saline (PBS), and dissected brains post‐fixed in the same solution for 24–48 h at 4 °C. Brains or hemispheres were sectioned coronally at 50 μm on a vibrating microtome (Leica, VT1000S).

To determine the onset of myelination, coronal hemi‐ or whole sections of control and cKO brains at postnatal days (P)6, P7 and P8 at the level of the striatum were stained for myelin basic protein (MBP) using immunohistochemistry. To determine the degree of myelination of SNAP25 cKO or Kir2.1‐IUE axons, coronal sections of P14 brains at the level of the corpus callosum, striatum, primary motor cortex (M1) and/or the site of electroporation were immunohistochemically stained for MBP. To determine Node of Ranvier (NoR) length, coronal sections of P14 brains at the level of the corpus callosum were immunohistochemically stained for both Caspr and MBP as well as anti‐RFP. To determine Olig2 and CC1 expression across development, sections containing M1 and striatum were double‐ stained immunofluorescently for Olig2 and CC1.

For detailed description of our immunohistochemical procedures see our previous publications (Hoerder‐Suabedissen et al. 2009, 2018a). Briefly, free‐floating sections were blocked with 2% donkey or goat serum and 0.2% Triton‐X100 in 0.1 m PBS for 2 h, before incubation with primary antibody (rat anti‐MBP (1 : 500; ab7349; Abcam) and rabbit anti‐DsRed (1 : 500; TaKaRa 632496; Clontech) or rat anti‐MBP (1 : 500; ab7349; Abcam) and chicken anti‐RFP (1 : 500; 600‐901‐379; Rockland) and rabbit anti‐Caspr (1 : 2000; ab34151; Abcam) for 48 h, or mouse anti‐CC1 (1 : 500, ab16794; Abcam) and rabbit anti‐Olig2 (1 : 200, AB9610; Millipore) for 24 h at 4 °C. Sections were washed in 0.1 m PBS before incubating with the secondary antibodies [MBP only: donkey anti‐rat AlexaFluor488 (1 : 500, A21208; Molecular Probes), and donkey anti‐rabbit AlexaFluor568 (1 : 500, A10042; Invitrogen); NoR: goat anti‐rabbit AlexaFluor488 (1 : 500, A11034; Molecular Probes), goat anti‐chicken AlexaFluor568 (1 : 500, A11041; Invitrogen), goat anti‐rat biotinylated (1 : 250, BA‐9400; Vector Laboratories), Olig2/CC1: donkey anti‐rabbit AlexaFluor488 (1 : 500, A21206; Molecular Probes) and donkey anti‐mouse biotinylated (1 : 100, ab7060; Abcam) in blocking solution at room temperature (RT) for 2 h. When biotinylated antibodies were used, sections were additionally incubated with streptavidin‐cy5 (1 : 200; Molecular Probes) for 2 h at RT. All the sections were counterstained with 4,6‐diamidine‐2‐phenylindole dihydrochloride (DAPI, 1 : 1000; Invitrogen) and mounted with Fluorsave (Millipore).

### Image analysis

#### Onset of myelination

Confocal images of striatum were taken with 20× objective for MBP‐stained sections (*n *=* *2–3 per brain) for both LV control and SNAP25 cKO brains at P7, P8 and P14. All images for analysis were taken with identical settings. The mean background intensity was calculated for all images across the ages using small ROIs devoid of tdTomato^+^ or MBP^+^ fibres. Pixels with intensity 2 SD above this background mean were taken to be MBP^+^, and the percentage of each image covered by such suprathreshold pixels was calculated.

#### MBP signal intensity and area

Confocal images of corpus callosum, striatum and either the electroporation site or M1 were taken using 40× objectives, for the MBP/dsRed‐stained sections (*n *=* *3 per brain) for *in utero* electroporated control and Kir2.1 mice, LVI SNAP 25cKO and control brains, and LV SNAP25 cKO and control brains. Image analysis was performed using Fiji (Schindelin et al. 2012). For callosal analysis, the tdTomato signal was binarised and used to define regions of interest (ROIs) in which the mean MBP intensity was measured. Outliers were identified (and discarded from further analysis) by measuring the MBP signal intensity in a 200 × 125 PixBox in a region without tdTomato signal. Images > 1 SD from mean MBP intensity of all ‘boxes' were discarded from further analysis. As such regions without any tdTomato signal could not be identified in LV brains, they therefore were not analysed for MBP intensity in the callosum.

For electroporation site/M1 analysis, the MBP channel was binarised and used to define ROIs. The area of the binary signal was measured as an indication of the amount of myelinated projections in this region, and the mean MBP intensity was measured in these ROIs as an indication of the amount of myelin. The amount of MBP at the level of cortical LV was minimal or absent at P14, and images were thus not further analysed.

For striatal analysis, the tdTomato signal was binarised and used to generate ROIs in which intensity of MBP signal was measured. As almost the entire striatum is densely innervated by LV tdTomato^+^ axon terminals by P14, we quantified MBP signal in LV SNAP25 cKO and control brains only in regions containing myelinated fibres (in this case, the MBP signal was thresholded and used to generate ROIs, in which the un‐thresholded MBP^+^ signal intensity was quantified).

#### Olig2/CC1 quantification

Manual cell density analysis was done using imagej (NIH), and conducted blind to genotype. The number of Olig2^+^ and CC1^+^ cells was detected using noise tolerance ranges 100–120 and 80–100, respectively, combined with JACoP plugin (Cordeliéres & Bolte, 2009).

#### Node of Ranvier length

For IUE, *Ntsr1‐Cre;Ai14;Snap25* ^*fl/fl*^ and *Rbp4‐Cre;Ai14;Snap25* ^*fl/fl*^ *,* and their respective control brains, confocal z‐stacks (interval 0.482 μm for 1 μm optical slice) and tiled images of the corpus callosum were acquired using a 63× objective from the Caspr, MBP and tdTomato/dsRed‐stained sections. In total, images for *in utero* electroporated control and Kir2.1 mice were acquired from 68 sections (*n *=* *6 brains) and 42 sections (*n *=* *3 brains), respectively. For LVI SNAP25 control and cKO mice, images were acquired from 38 sections (*n *=* *4 brains) and 46 sections (*n *=* *5 brains), respectively, at P14. For LV SNAP25 control and cKO mice, images were acquired from 23 and 28 sections (*n *=* *3 and 7 brains), respectively. Images were analysed using Fiji, utilising a method adapted from Arancibia‐Carcamo et al. (2017).

A node was selected when a tdTomato projection could be seen overlaid by the Caspr‐labelled paranodes and flanked by MBP to ensure that only nodes on electroporated projections were measured (*n *=* *116 for control and *n *=* *66 for Kir2.1; *n *=* *46 for control and *n *=* *35 for LVI SNAP25 cKO; and *n *=* *33 for control and *n *=* *58 for LV SNAP25 cKO). A maximum intensity projection was then generated for each node (maximum of five interleaved confocal slices). A line intensity profile was measured over the grey‐scale region of Caspr‐labelled paranodes (thickness = 5, slightly thinner than the paranode‐labelling for most nodes). The size of the node was then calculated using the MATLAB script kindly provided by David Attwell (Arancibia‐Carcamo et al. 2017). Briefly, the maximum intensity for each paranode is calculated, the minimum intensity between paranodes is subtracted from this, and the half‐maximum identified. Distance between half‐maxima of both paranodes along the line‐intensity profile is calculated.

### Scanning electron microscopy

Animals for scanning electron microscopy (SEM) were anaesthetised with 0.5 mL pentobarbitone (20% w/v, animal care) and transcardially perfused for 12 min using a perfusion pump with flow rate set to < 50% of maximum cardiac output. Perfusion started with 5–10 mL of PBS (0.1 m PBS, pH 7.4) followed by fixative (2.5% glutaraldehyde; EM grade from EMS) and 2% paraformaldehyde (same source) in 0.1 m PBS pH 7.4) at room temperature. After the perfusion, animals were left for 2 h at room temperature before dissection. Spinal cords were sent to Bio EM Facility of Ecole Polytechnique Federale de Lausanne (EPFL), where they were embedded in 5% agarose (Bioline, in PBS) for cutting with a vibrating microtome (VT1000S; Leica Systems). Sections of 80 μm were washed three times for 10 min each in cacodylate buffer (0.1 m, pH 7.4; Electron Microscopy Scientific) before postfixing on ice in freshly prepared 1.5% potassium ferrocyanide, 0.13 M cacodylate buffer and 2% osmium tetroxide (diluted in PB, pH 7.2; Agar Scientific) for 1 h. Sections were washed five times for 3 min each in water between all following steps. Sections were stained in filtered thiocarbohydrazide (1%; Sigma Aldrich) for 20 min before being transferred into 2% osmium tetroxide (Agar Scientific) for a further 30 min, followed overnight in 1% uranyl acetate at 4 °C. After this, sections were washed in 50 °C water three times for 10 min followed by a contrast stain with Walton's lead aspartate solution (10 mL water heated to 50 °C, 0.04 g aspartic acid, 0.066% lead nitrate, pH 5.0) at 50 °C for 2 h. During the following three wash steps, water temperature was gradually lowered from 50 °C to room temperature before dehydration was started using graded alcohol series of 5 min each (1 × 50, 1 × 70, 1 × 96, 2 × 100%). After dehydration, sections were transferred to increasing concentrations of epoxy resin (Durcupan; Sigma‐Aldrich) in ethanol, and left in 100% resin overnight. Sections were placed between glass slides coated with mould release agent (Glorex, Switzerland) and for 24 h at 60 °C, before cutting semi‐thin sections (500 nm) using an ultramicrotome (UC7; Leica Microsystems). Semi‐thin sections were toluidine blue‐stained for 4 min at 60 °C and imaged using a transmitted‐light microscope (Zeiss Axioskop 2) to locate the precise region for ultra‐thin sectioning. Ultrathin sections of the area that contains Rbp4^+^ corticospinal fibres in the spinal cord were prepared, collected onto silicon wafers of 10‐mm diameter and imaged using a scanning electron microscope (Merlin, Zeiss NTS) with a beam voltage of 1.8 kV and pixel size of 8 nm. The entire corticospinal tract was imaged by tiling multiple images using custom software (SBEM Image, Benjamin Titze, FMI, Basel, Switzerland).

We quantified g‐ratios from SEM images in the region of the dorsal corticospinal tract; the experimenter was blind to genotype at the time of image analysis. Quantification was across all dorsal column axons that contained the manipulated *Rbp4‐Cre;Ai14;Snap25* ^*fl/fl*^ projections; as 15% of LV NeuN^+^ neurons at P6 are Cre^+^ (Hoerder‐Suabedissen et al. 2018b), we assume that a similar proportion of dorsal column axons are Cre^+^ . We would have preferred to determine the g‐ratio specifically of cKO vs. control *Rbp4‐Cre;Ai14* tdTomato‐labelled axons in the dorsal column and compare these with the tdTomato‐negative projections, but we could not find fixation conditions under which myelin was well preserved and antigenicity for the detection of tdTomato remained (various concentrations and fixation lengths were tested). After several attempts, we opted for fixation optimised for myelin preservation (see above), and quantified the g‐ratio across all axons in a region of the spinal cord containing the dorsal column where the tdTomato‐labelled axons were observed before embedding. We used scanning electron microscopy to obtain a larger field of view, ensuring correct identification of the entire dorsal column. The g‐ratios were quantified for all myelinated axons in a 32.5 μm × 42.5 μm box, the largest rectangular ROI that is entirely contained within the dorsal corticospinal tract.

### Statistics

Analysis was performed on data from at least *n *=* *2 brains per group (see Supporting Information Table S1 for a breakdown of brains used for each age and genotype) and for each analysis a minimum of two sections per brain. Data are shown as mean ± SEM. Significance was determined using unpaired 2‐tailed Student's *t*‐tests with unequal variances, anova with post‐hoc Bonferroni test (Olig2/CC1 data) or Mann–Whitney rank sum test (Node of Ranvier data). Statistical analysis on NoR length measurements was on the NoR individual measurements, not the per‐brain averages.

## Results

**Figure 1:**
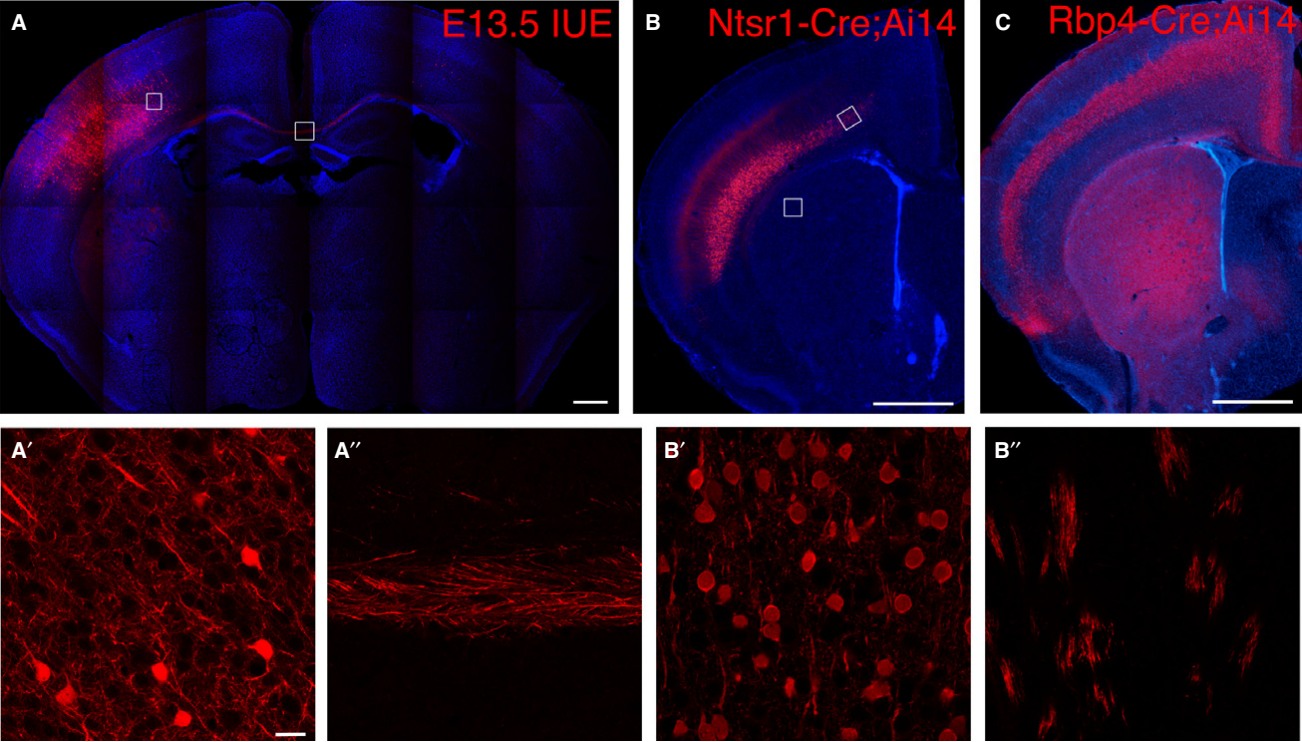
Distribution of cortical cells labelled by E13.5 *in utero* electroporation, *Ntsr1‐Cre;Ai14* or *Rbp4‐Cre;Ai14*. (A) Laser scanning, tiled confocal microscope image of a P14 brain section after IUE at E13.5. Note the unilateral electroporation site and the callosal fibres. Approximate location of higher magnification view of the cortex and callosum are indicated with white boxes, and shown in (A′) for cortex and (A″) for callosum. (B) Epifluorescent microscope image of P14 *Ntsr1‐Cre;Ai14* brain, labelling mostly LVIa cells in the cerebral cortex (LVI control). These cells form sparse callosal and denser striatal projections, but they are too faint to be visible. Locations of higher magnification view of the cortex and striatum are indicated with white boxes, and are shown in (B′) for cortex and (B″) for striatum to demonstrate the presence of red fibres. (C) Epifluorescent microscope image of P14 *Rbp4‐Cre;Ai14* brain, labelling mostly LV cells in the cerebral cortex (LV control). Note the strong callosal and striatal projections from this cell population. Scale bars: 1 mm (A–C), 20 μm (A′,A″,B′,B″). *IUE*,* in utero* electroporation.

All experiments in this study were conducted in mice. We reduced electrical excitability of a mixed LV and LVI population by *in utero* electroporation of Kir2.1 at E13.5 (Fig. 1A), or ablated calcium‐dependent vesicle release in two distinct cortical cell populations (LV in *Rbp4‐Cre* and LVI in *Ntsr1‐Cre*, see Fig. 1C,B) by conditional knock‐out of *Snap25*. Some of the LV axons are the largest and longest, with projections all the way to the spinal cord, which are heavily myelinated. However, LV SNAP25 cKO axons show signs of axonal degeneration from P22 onwards (Hoerder‐Suabedissen et al. 2018b). LVI cells labelled in *Ntsr1‐Cre;Ai14;Snap25* ^*fl/fl*^ brains have a much slower axonal degeneration, only obvious by 2 months of age (Hoerder‐Suabedissen et al. 2018b). We therefore used both strains for many of our analyses to ensure that any effects observed are not purely preliminary to axon degeneration. In both *Rbp4‐Cre;Ai14;Snap25* ^*fl/fl*^ and *Ntsr‐Cre;Ai14;Snap25* ^*fl/fl*^ mice, onset of Cre‐expression, and therefore knock‐down of SNAP25 occurs during late embryonic development (Hoerder‐Suabedissen et al. 2018b) and well before the onset of myelination in the murine brain (Foran & Peterson, 1992) and stays constantly expressed through life.

### Onset of myelination is not affected by Snap25 cKO

**Figure 2:**
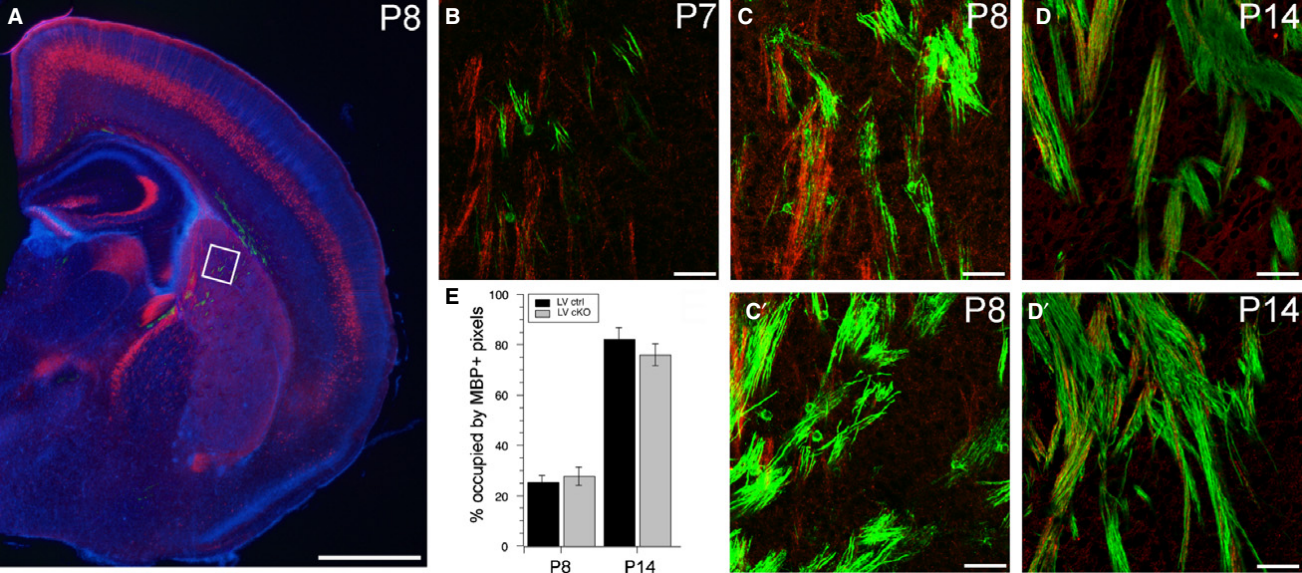
Onset of myelination is not affected by SNAP25 cKO. (A) Epifluorescent microscope image of a LV control (red) mouse brain counterstained with DAPI (blue) and immunofluorescently stained for MBP (green) at the approximate position used to document onset of MBP expression in all brains. White box indicates the region from which higher magnification panels are taken, and in which MBP signal intensity was quantified. (B) Laser scanning confocal microscope image of tdTomato (red) and MBP (green) overlay image of a LV control brain at P7. (C,C′) tdTomato (red) and MBP (green) overlay images of LV control (C) and LV SNAP25 cKO (C′) striatum at P8. (D,D′) tdTomato (red) and MBP (green) overlay images of LV control (D) and LV SNAP25 cKO (D′) striatum at P14. (E) Quantification of the percentage area covered by MBP ^+^ suprathreshold pixels (see Materials and methods for definition) at P8 and P14 in LV control and SNAP25 cKO striatal images. There was no significant difference between ctrl and cKO at the ages tested. Scale bars: 1 mm (A), 50 μm (B–D,C′,D′). ctrl, control.

For LV SNAP25 cKO (*Rbp4‐Cre;Ai14;Snap25* ^*fl/fl*^ *)* and controls (*Rbp4‐Cre;Ai14* and *Rbp4‐Cre;Ai14;Snap25* ^*fl/+*^), onset of myelination in the brain was assessed using MBP immunofluorescence (at least *n *=* *2 brains per genotype). At P6, no MBP staining was observed in the brain, at the level of striatum and primary somatosensory cortex (data not shown). At P7, in all genotypes, a few short fibres, typically at the junction of white matter and striatum or in the striatum and internal capsule were MBP^+^ (see Fig. 2B for LV control). At this age, the MBP^+^ signal was sufficiently sparse, so it was often possible to identify individual myelinating oligodendrocytes, typically ensheathing several distinct axons. The majority of myelinated fibres in the striatum are not tdTomato^+^ in LV control brains at P7. More fibres are myelinated for longer stretches by P8, still predominantly in the striatum and internal capsule, but also emerging in the cortical white matter (Fig. 2A,C). The majority of tdTomato^+^ axons do not overlap with regions of MBP^+^ signal. By P14, all thick fibre tracts in the striatum overlap with areas of strong MBP^+^ signal, and at this age most tdTomato^+^ fibres lie within regions of MBP^+^ signal and are presumed to be myelinated (Fig. 2D). In cortex, strong MBP^+^ signal is evident in the white matter, with sparser label in L6a, which extends approximately up to the lower edge of LV (data not shown). No difference was observed in the location of myelination or onset of myelination between LV SNAP25 cKO and control brains. The percentage of image area covered by immunofluorescence signal for MBP was not significantly different at P8 or P14 in LV control and SNAP25 cKO brains [P8: 25.2% ± 2.7 (ctrl, *n *=* *4 brains) vs. 27.7% ± 3.7 (cKO, *n *=* *2 brains), *P *=* *0.39; P14: 82.1% ± 4.6 (ctrl, *n *=* *3 brains) vs. 76.0% ± 7.6 (cKO, *n *=* *3 brains), *P *=* *0.13; Fig. 2E], but shows the clear trend towards increased area covered from P7 to P14 in LV control brains. Control tissue at P8 was a mix of *Rbp4‐Cre;Ai14* and *Rbp4‐Cre;Ai14;Snap25 ^fl/+^* genotypes, with the *Rbp4‐Cre;Ai14;Snap25 ^fl/+^* animals being littermates to the LV SNAP25 cKO pups used for this timeline. A qualitatively similar overall timeline of development (no MBP signal at P6, a few MBP^+^ oligodendrocytes in striatum at P7, but typically not overlapping the tdTomato^+^ fibres, and almost complete myelination in striatum at P14) was also observed for *Ntsr1‐Cre;Ai14;Snap25 ^fl/fl^* (LVI SNAP25 cKO) and control brains (data not shown).

### Altered levels of MBP following Snap25 cKO or Kir2.1 IUE

The callosum, in particular, is a structure containing axons with diverse laminar origins, and with a wide range of axon diameters. A considerable overlap in axon calibre exists between myelinated and unmyelinated axons (Sturrock, 1980). We therefore initially confirmed that tdTomato‐labelled axons in LV control (*Rbp4‐Cre;Ai14*) and LVI control (*Ntsr1‐Cre;Ai14)* brains as well as the control‐tdTomato *in utero* electroporation‐labelled axons within the corpus callosum are myelinated by P14. For all three axon types there are tdTomato^+^ axons overlaid by MBP^+^ myelin sheaths, but many others that are not myelinated (data not shown). We conducted all subsequent analyses of corpus callosum at P14, as LV SNAP25 cKO axons show the first signs of degeneration by P22 (Hoerder‐Suabedissen et al. 2018b).

**Figure 3:**
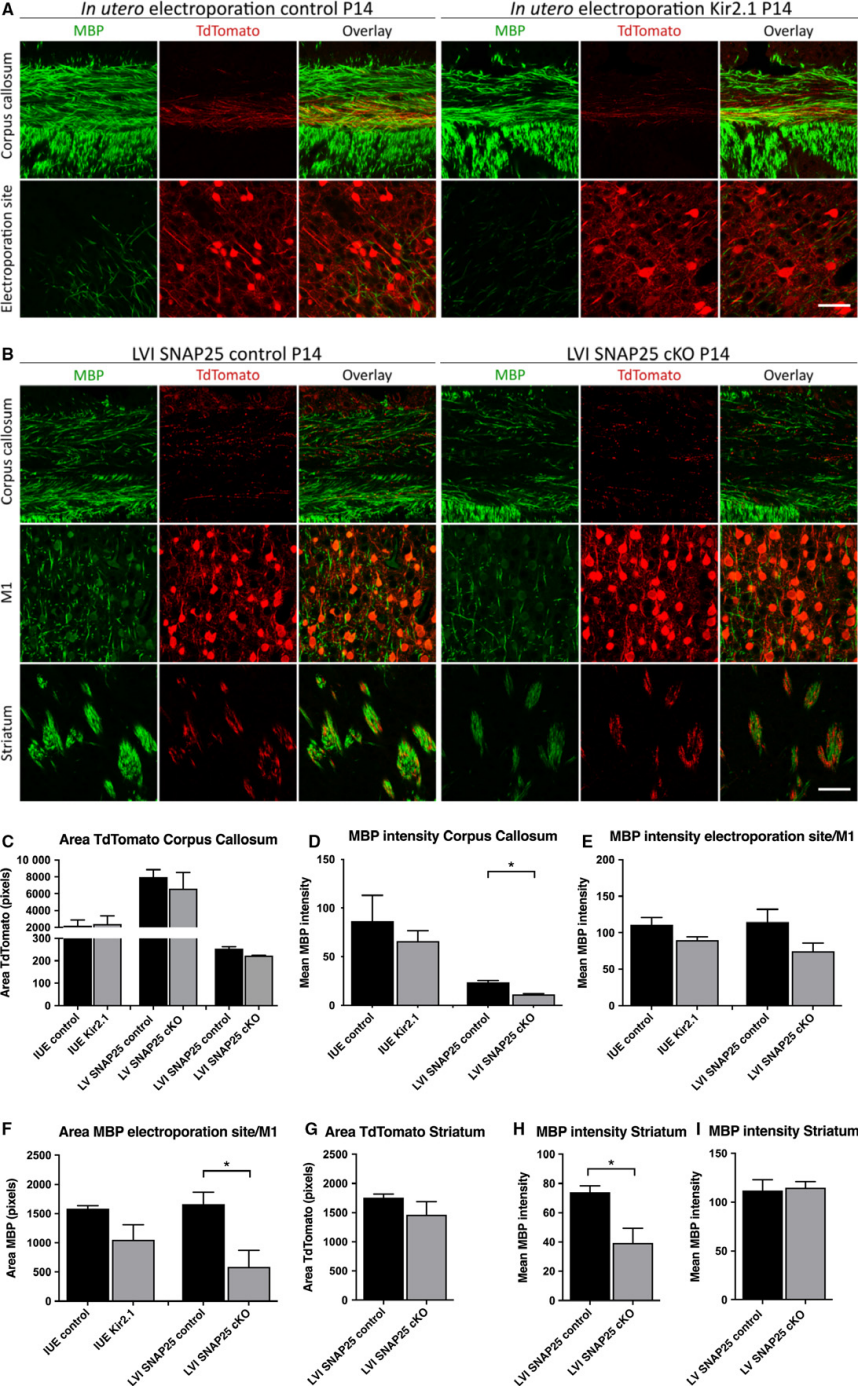
Amount of MBP is decreased in LVI SNAP25 cKO but not after Kir2.1 *in utero* electroporation at P14. (A) Laser scanning confocal microscope images of the corpus callosum and electroporation site of P14 control and Kir2.1 in *in utero* electroporated brains, stained for MBP (green) and tdTomato (red). (B) Images of the corpus callosum, M1 and striatum of P14 control and LVI SNAP25 cKO brains, stained for MBP (green) and tdTomato (red). (C) Quantification of the area of tdTomato^+^ projections in the corpus callosum of IUE, LV SNAP25 cKO, LVI SNAP25 cKO (grey) and their respective controls (black), suggesting no significant difference in the amount of labelled fibres crossing the midline between manipulated and control brains. (D) Quantification of mean MBP intensity in the corpus callosum in a region of interest (ROI) determined by tdTomato‐positive projections. There was no significant difference between the mean MBP intensity in *in utero* electroporated brains, but the mean MBP intensity for *Ntsr1‐Cre;Ai14;Snap25* ^*fl/fl*^ (LVI SNAP25 cKO) brains is significantly lower than for age‐matched controls. (E) Quantification of the mean MBP intensity in the electroporation site or M1 in an ROI defined by MBP^+^ projections, which is not significantly different between controls and *in utero* Kir2.1‐electroporated or LVI SNAP25 cKO brains. This quantification was not done for LV SNAP25 cKO brains, as there is barely any myelin in LV at this age, even in control brains. (F) Quantification of the area labelled by MBP in the electroporation site or M1. The area labelled by MBP for LVI SNAP25 cKO brains is significantly lower than for age‐matched controls, suggesting that although each myelinated fibre may be myelinated to the same degree as in controls, the overall number of processes myelinated is significantly lower. (G) Quantification of area of tdTomato‐positive projections in the striatum of LVI SNAP25 cKO and control brains, which was not significantly different. (H) Quantification of the mean MBP intensity in an ROI of the striatum defined by tdTomato^+^ projections from LVI cells. (I) Quantification of mean MBP intensity in ROI of the striatum defined by regions with MBP ^+^ axon bundles, most of which colocalised with tdTomato^+^ axon bundles from LV cells. The MBP intensity in LVI but not LV SNAP25 cKO brains was significantly lower than in control brains. All measures are mean ± SEM. *Significance of *P* < 0.05 using 2‐tailed Student's *t*‐tests with unequal variance. Scale bars:  50 μm.

To investigate whether reduced neuronal excitability or abolished vesicle fusion affects myelination in the mouse corpus callosum in which SNAP25 cKO and normal fibres are closely intermingled, we imaged MBP and tdTomato fluorescence‐immunoreactivity and measured the intensity of the MBP signal and amount of electroporated axons myelinated at P14 in Kir2.1‐IUE, and LVI SNAP25 cKO brains and their respective controls. At P14, myelination is still in progress (Foran & Peterson, 1992), but myelinated tdTomato^+^ axons are present in the corpus callosum (see above). The area of corpus callosum occupied by tdTomato^+^ axons was similar for Kir2.1‐IUE and tdTomato‐IUE (control) brains [2414.09pix ± 947.39 (Kir2.1, *n *=* *2) vs. 2215.51pix ± 646.94 (ctrl, *n *=* *3), mean ± SEM], the LVI SNAP25 cKO and control brains [222.98pix ± 1.59 (cKO, *n *=* *3) vs. 254.89pix ± 6.67 (ctrl, *n *=* *3), mean ± SEM] and the LV SNAP25 cKO and control brains [6592.94pix ± 1939.1 (cKO, *n *=* *3), vs. 7967.1pix ± 881.7 (ctrl, *n *=* *3) mean ± SEM.; Fig. 3C]. However, it did differ markedly between control IUE, and LVI control brains, with LVI control brains showing *many* fewer labelled axons in the corpus callosum, despite the much higher number of labelled cells achieved with LVI labelling of the entire cortical mantle, compared with a unilateral electroporation site restricted to S1. This may suggest that the majority of callosal axons in IUE brains are LV‐derived. Overall, LV control brains had the highest density of tdTomato^+^ fibres in the callosum, but also dense tdTomato^+^ processes in all other structures surrounding the corpus callosum.

When comparing the mean MBP signal intensity in the region of tdTomato^+^ fibres in the corpus callosum, a significant reduction in MBP signal intensity in LVI SNAP25 cKO brains compared with controls was evident [11.34 ± 0.66 (cKO, *n *=* *3) vs. 23.63 ± 1.90 (ctrl, *n *=* *3), mean ± SEM, *P *=* *0.015, Fig. 3D]. No such effect was observed following Kir2.1 IUE in the corpus callosum [66.08 ± 10.56 (Kir2.1, *n *=* *2) vs. 86.45 ± 26.58 (ctrl, *n *=* *3), mean ± SEM, *P *=* *0.54, Fig. 3D]. For LV, the corpus callosum was not analysed, as we could not identify regions without any red fibres that could be used to normalise/discard images with inappropriate background.

A similar reduction in MBP signal intensity was observed in the striatum of LVI SNAP25 cKO compared with controls [39.4 ± 10.1 (cKO, *n *=* *4) vs. 74 ± 4.4 (ctrl, *n *=* *3), mean ± SEM, *P *=* *0.03, Fig. 3H], whereas the total area covered by tdTomato^+^ signal was again unchanged between the two conditions [1463.6pix ± 224.7 (cKO, *n *=* *4) vs. 1760.7pix ± 55.6 (ctrl, *n *=* *3), mean ± SEM, *P *=* *0.28, Fig. 3G]. For LV SNAP25 cKO and control brains, no significant difference in the area of striatum covered by MBP^+^ signal was observed at P14 (see section on MBP development above), and the mean intensity in the region covered by MBP^+^ signal was also similar [115 ± 6 (cKO, *n *=* *3) vs. 112 ± 11 (ctrl, *n *=* *3), mean ± SEM, *P *=* *0.72; Fig. 3H]. The striatum or the cerebral peduncle was not analysed for Kir2.1 IUE brains due to the sparsity of labelled axons traversing this region.

We also investigated the mean MBP signal intensity in regions with MBP, and total area covered by MBP signal in primary motor cortex (M1) or the electroporation site (usually S1). The total area of each image covered by MBP‐positive structures was significantly reduced for LVI SNAP25 cKO brains compared with controls [589.6pix ± 284.4 (cKO, *n *=* *4) vs. 1661.3pix ± 203.7 (ctrl, *n *=* *3), mean ± SEM, *P *=* *0.029] but not Kir2.1 IUE brains (1053pix ± 259 (Kir2.1, *n *=* *3) vs. 1582.8pix ± 52 (ctrl, *n *=* *3), mean ± SEM, *P *=* *0.17; Fig. 3F]. LV SNAP25 cKO and control brains were not analysed for M1 cortex, as the MBP immunofluorescent signal has barely reached the lower edge of cortical LV at this age. Because of the differences in area covered by MBP^+^ signal, we determined the mean MBP signal intensity in regions of MBP signal (to avoid bias as a result of overall different numbers of myelinated structures). This was not significantly different for LVI SNAP25 cKO brains or Kir2.1 IUE compared with their respective controls [74.8 ± 11 (cKO, *n *=* *4) vs. 114.7 ± 17.5 (ctrl, *n *=* *3), *P *=* *0.14 and 89.9 ± 4.6 (Kir2.1, *n *=* *3) vs. 111.2 ± 9.7 (ctrl, *n *=* *3), *P *=* *0.15, respectively, Fig. 3E]. This may suggest that each myelinated fibre is still normally myelinated (no difference in intensity) but that overall fewer structures are myelinated in LVI SNAP25 cKO brains.

### Few differences in oligodendrocyte maturation after SNAP25 cKO

To determine whether observed differences in myelination as assessed by MBP immunofluorescence might be the result of delayed maturation, we used Olig2 and CC1 as markers of the oligodendrocyte lineage to quantify oligodendrocyte precursor cells (OPCs), pre‐oligodendrocytes and mature oligodendrocytes at P8 (after the onset of myelination), at P14 and at P21. Olig2 is expected to be expressed in all cells of the oligodendrocyte lineage, irrespective of the state of differentiation, but expression levels decline in myelinating oligodendrocytes (Kitada & Rowitch, 2006). The CC1 monoclonal‐antibody is commonly used specifically to label mature oligodendrocyte cell bodies (Bhat et al. 1994; Fuss et al. 2000; Messersmith et al. 2000), as it is not seen in dividing cells (Kitada & Rowitch, 2006).

**Figure 4:**
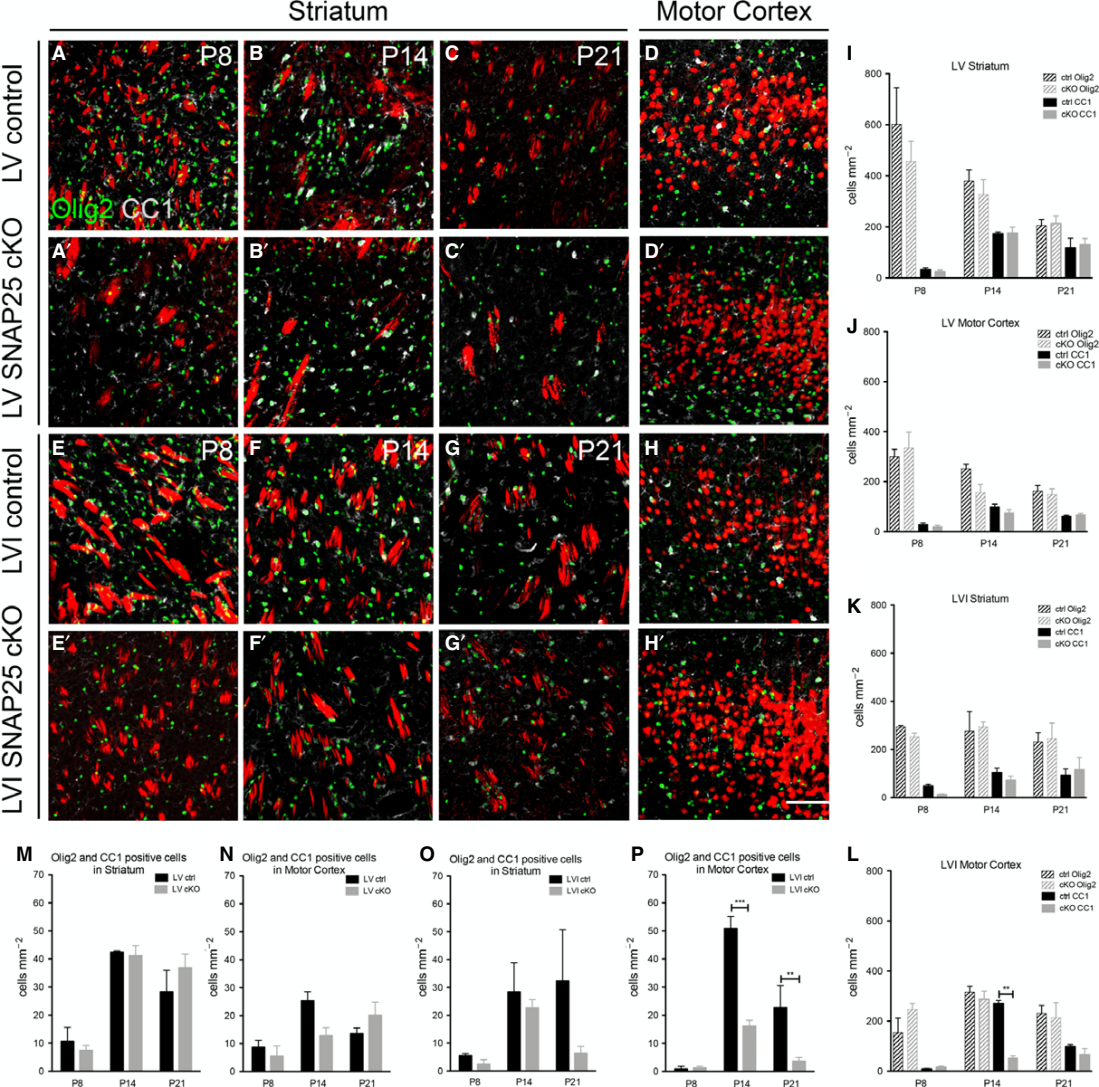
Oligodendrocyte maturation as measured by CC1 expression is reduced after LVI SNAP25 cKO, but unaffected by LV SNAP25 cKO. (A‐C) Confocal images of striatum of LV control brains labelled with anti‐Olig2 (green) and anti‐CC1 (white) immunofluorescence at P8, P14 and P21. (A′–C′) Confocal images of striatum of LV SNAP25 cKO brains labelled with anti‐Olig2 (green) and anti‐CC1 (white) immunofluorescence at P8, P14 and P21. (D) Confocal image of LV of cerebral cortex of LV control brains labelled with anti‐Olig2 (green) and anti‐CC1 (white) immunofluorescence at P14. (D') Confocal image of LV cortex of LV SNAP25 cKO brains labelled with anti‐Olig2 (green) and anti‐CC1 (white) immunofluorescence at P14. (E‐G) Confocal images of striatum of LVI control brains labelled with anti‐Olig2 (green) and anti‐CC1 (white) immunofluorescence at P8, P14 and P21. (E′–G′) Confocal images of striatum of LVI SNAP25 cKO brains labelled with anti‐Olig2 (green) and anti‐CC1 (white) immunofluorescence at P8, P14 and P21. (H) Confocal image of LVI of cerebral cortex of LVI control brains labelled with anti‐Olig2 (green) and anti‐CC1 (white) immunofluorescence at P14. (H') Confocal image of LVI cortex of LVI SNAP25 cKO brains labelled with anti‐Olig2 (green) and anti‐CC1 (white) immunofluorescence at P14. (I) Quantification of Olig2^+^ and CC1^+^ cells in striatum of LV control and SNAP25 cKO brains across the three ages. (J) Quantification of Olig2^+^ and CC1^+^ cells in motor cortex (at the level of LV) of LV control and SNAP25 cKO brains across the three ages. (K) Quantification of Olig2^+^ and CC1^+^ cells in striatum of LVI control and SNAP25 cKO brains across the three ages. (L) Quantification of Olig2^+^ and CC1^+^ cells in motor cortex (at the level of LVI) of LVI control and SNAP25 cKO brains across the three ages. There was a significant reduction in the density of CC1^+^ cells at P14, but not at any other age, in LVI SNAP25 cKO brains compared with controls. (M) Quantification of Olig2^+^ CC1^+^ double‐labelled cell density in the striatum of LV control or SNAP25 cKO brains. (N) Quantification of Olig2^+^ CC1^+^ double‐labelled cell density in motor cortex of LV control or SNAP25 cKO brains. (O) Quantification of Olig2^+^ CC1^+^ double‐labelled cell density in the striatum of LVI control or SNAP25 cKO brains. (P) Quantification of Olig2^+^ CC1^+^ double‐labelled cell density in motor cortex of LVI control or SNAP25 cKO brains. There was a significant reduction in double‐labelled cells in SNAP25 cKO brains compared with controls at P14 and P21, but not at P8. Scale bar: 150 μm (applies to all panels). **P < 0.01, ***P < 0.001.

Marker expression was studied in the layer of the cortex where Cre‐expressing cell bodies were found, as well as the striatum. Very few CC1^+^ cells were detected by immunohistochemistry in M1 or striatum of LV and LVI SNAP25 cKO and control brains at P8. In contrast, Olig2^+^ cells were abundant at this age both in M1 and striatum (Fig. 4). The density of CC1^+^ cells increased with increasing age, both in striatum and cortex, as did the density of CC1^+^Olig2^+^ double‐positive cells.

There were no significant differences between cKO and control brains in striatum of LV or LVI brains when comparing Olig2^+^ cell density, CC1^+^ cell density or density of Olig2^+^ CC1^+^ double positive cells at any age studied (see Fig. 4). In contrast, in M1, the density of CC1^+^ cells and of Olig2^+^CC1^+^ double‐positive cells was significantly lower in LVI SNAP25 cKO brains at P14 (CC1^+^ cells – 270 ± 23 cells mm^−2^ (LVI ctrl, *n *=* *3) vs. 53 ± 16 cells mm^−2^ (LVI cKO, *n *=* *4), *P *<* *0.01; Olig2^+^CC1^+^ double‐positive – 51 ± 7 cells mm^−2^ (LVI ctrl) vs. 16 ± 4 cells mm^−2^ (LVI cKO), *P *<* *0.001). By P21, the CC1^+^Olig2^+^ double‐positive cell density was still much lower in LVI cKO brain than in controls [23 ± 14 cells mm^−2^ (LVI ctrl) vs. 4 ± 3 cells mm^−2^ (LVI cKO), *P *<* *0.01]. There were no significant differences in M1 at P8 in LVI SNAP25 cKO brains compared with controls, and no differences in motor cortex of LV SNAP25 cKO brains at any age.

Thus, the differences in MBP levels seen in LVI SNAP25 cKO compared with control brains at P14 are accompanied by differences in oligodendrocyte maturation at the same age, which subsequently do not appear to fully resolve. No differences in oligodendrocyte maturation were observed between LV SNAP25 cKO and control brains, which ties in well with the absence of any significant differences in MBP intensity or area covered between the two genotypes.

### Decreased NoR length after Kir2.1 IUE or SNAP25 cKO

NoR are essential for action potential conduction on myelinated fibres, and their spacing (internodal length) and width influence action potential conduction velocity and reliability. Moreover, the paranode region, containing Caspr cell adhesion molecules, is an essential attachment site for the myelin sheaths. We therefore studied NoR formation in the projections of SNAP25 cKO and Kir2.1 IUE cortical neurons and their respective controls.

Given the dense packing of axons in the corpus callosum, and the fact that they never lie perfectly straight and parallel to the plane of sectioning, we could not measure the internodal length.

**Figure 5:**
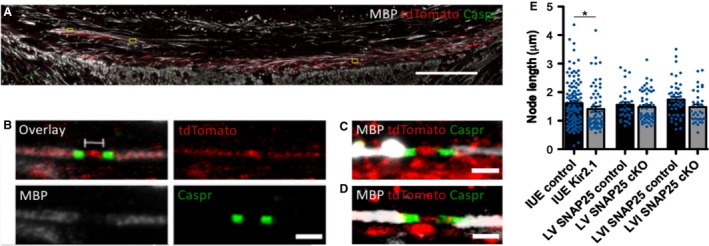
Node of Ranvier length reduction after SNAP25 cKO or Kir2.1 *in utero* electroporation compared with controls. (A) Confocal tiled images of the corpus callosum in an IUE control brain at P14. Myelin basic protein (MBP) is shown in white, tdTomato indicating electroporated fibres in red and Caspr in green. Yellow boxes outline measured nodes. (B) Example of a measured node of Ranvier in an IUE control brain, where a tdTomato^+^ axon is seen at the node, overlaid by the paranodes (Caspr) and flanked by myelin (MBP) at the internodes. (C) Example of a measured node of Ranvier in the corpus callosum of a P14 LV control brain. (D) Example of a measured node of Ranvier in the corpus callosum of a P14 LVI control brain. (E) Bar graphs showing the mean nodal length for Kir2.1 and control IUE, LVI SNAP25 cKO and control, and LV SNAP25 cKO and control. There was a significant difference in node length for Kir2.1 IUE brains compared with controls. Individual node measurements are indicated with blue dots overlying the bar graphs. Data given as mean ± SEM. Scale bars: 100 μm (A), 3 μm (B‐D). *P < 0.05 (Mann–Whitney rank sum test).

To measure the NoR length, we immunostained the IUE or SNAP25 cKO and control brains for MBP, tdTomato and Caspr, and imaged the corpus callosum with tiles and stacks at high magnification on a laser‐scanning confocal microscope. Only nodes where a tdTomato^+^ axon was overlaid by paranodes flanked by MBP were measured (see Fig. 5B). To measure the nodal length, we determined the maximum intensity of each paranode signal, and calculated the length between the half maxima of each paranode signal as an indication for paranode border, using the same methods as Arancibia‐Carcamo et al. (2017). For all three manipulations (Kir2.1 IUE, and LV or LVI SNAP25 cKO) there was a strong trend for significant reduction in NoR length. For Kir2.1 *in utero* electroporated brains, NoR were significantly shorter than in control electroporated brains [1.61 μm ± 0.07 (ctrl, *n *=* *116 nodes in six brains) vs. 1.41 μm ± 0.09 (Kir2.1, *n *=* *66 nodes in three brains), mean ± SEM, *P *=* *0.042, Mann–Whitney rank sum test; see Fig. 5E]. A strong trend towards shorter NoR was also observed in LVI SNAP25 cKO brains compared with controls [1.47 μm ± 0.06 (LVI ctrl, *n *=* *46 nodes in three brains) and 1.42 μm ± 0.09 (LVI cKO, *n *=* *35 nodes in five brains), *P *=* *0.051, Mann–Whitney rank sum test] and in the LV SNAP25 cKO brains compared with controls [1.56 μm ± 0.09 (LV ctrl, *n *=* *33 in three brains) and 1.48 μm ± 0.07 (LV cKO, *n *=* *58 in seven brains), *P *=* *0.051, Mann–Whitney rank sum test]. For LV and LVI SNAP25 cKO, not every section imaged contained quantifiable NoRs (hence the relatively smaller number of nodes analysed compared with the IUE brains). We searched for NoR in 46 and 38 sections from LVI SNAP25 cKO and control brains, but detected far fewer NoR in the cKO sections (35 nodes in 46 sections for cKO and 46 nodes in 38 sections for controls). Overall, half of LVI SNAP25 cKO sections contained no nodes that met our criteria, compared with less than one‐third of sections in control brains. For LV, we searched for NoR in 28 and 18 sections from SNAP25 cKO and control, respectively (58 nodes in 28 sections for cKO and 33 nodes from 18 sections for controls). This may suggest that SNAP25 cKO in LVI neurons partially suppresses myelination including NoR formation and is in agreement with the reduction in MBP signal reported above for LVI SNAP25 cKO but not LV SNAP25 cKO.

The reduction of NoR length observed after LV and LVI SNAP25 cKO or Kir2.1 IUE indicates that both decreased regulated vesicular release and decreased excitability of axons could affect the length of the NoR. Reduced vesicular release may additionally reduce overall levels of myelination, including formation of nodes, but this appears to be strongly cell type‐specific. The net effect of the change in NoR length is not immediately obvious – shorter nodes reduce the amount of energy required to propagate each action potential and increase the speed of conduction, but too short a node length will result in failure to conduct action potentials.

### Altered g‐ratio in dorsal column of LV Snap25 cKO spinal cord

**Figure 6:**
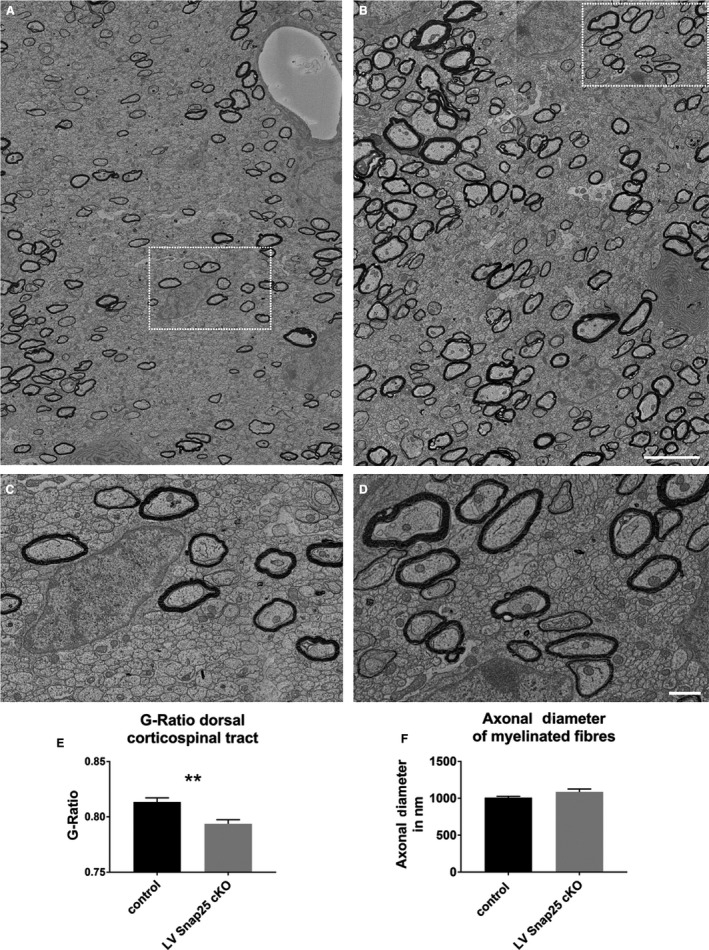
Reduced g‐ratio in LV SNAP25 cKO dorsal spinal columns despite normal axon diameter. (A) Tiled scanning electron microscope (SEM) image of the g‐ratio quantification region of one side of dorsal spinal column in LV control mice at P18. A higher magnification view of the boxed region is shown in (C). (B) Tiled SEM image of the g‐ratio quantification region of one side of dorsal spinal column in LV SNAP25 cKO spinal cord at P18. A higher magnification view of the boxed region is shown in (D). (E) The average g‐ratio of LV SNAP25 cKOs (*n* = 4) shows a significant reduction in comparison to the g‐ratio of controls (*n* = 5, *P* = 0.0072). (F) Axonal diameter of myelinated fibres was not significantly different between controls and LV SNAP25 cKOs. Scale bars: 5 μm (A,B) 1 μm (C,D).

Another measure of myelin integrity is the g‐ratio, and whether all myelin rings are appropriately compacted. To study this, we opted for electron microscopic imaging of P18 LV SNAP25 cKO and control spinal cords, a time point at which myelin has had more than 2 weeks to mature after birth, but which is still 5 days away from the time of demonstrable axon disintegration in LV SNAP25 cKO spinal cords (Hoerder‐Suabedissen et al. 2018b). We would have preferred to determine the g‐ratio of tdTomato‐labelled SNAP25 cKO vs. LV control axons in the dorsal column but could not find fixation conditions under which myelin was well preserved and antigenicity for tdTomato was retained. Thus, we opted for fixation optimised for myelin preservation (2% paraformaldehyde and 2.5% glutaraldehyde), and quantified the g‐ratio across all axons in a region of the spinal cord containing the dorsal column (Fig. 6). We used scanning electron microscopy to obtain a larger field of view, ensuring correct identification of the entire dorsal column. The g‐ratios were quantified in a 32.5 μm × 42.5 μm box which was entirely contained within the dorsal corticospinal tract. LV SNAP25 cKO mice had a significantly reduced mean g‐ratio (0.794 ± 0.0036, *n *=* *2192 axons measured in four spinal cords) compared with controls (0.813 ± 0.0037, *n *=* *2302 axons measured in five spinal cords; *P *=* *0.0072; Fig. 6). A reduced g‐ratio corresponds to an increase in the amount of myelin surrounding an axon of a given diameter. The g‐ratio is known to decrease with increasing axon size, but there was no significant difference between axon diameters measured in the control (1008 nm ± 19) and SNAP25 cKO (1086 nm ± 40, *P *=* *0.127) spinal cord. There was an overall shift in the distribution of g‐ratios towards lower numbers across a range of values, but the correlation of axon diameter and g‐ratio was not significantly altered, suggesting an effect across a range of axon diameters.

## Discussion

We compared levels of myelin as measured by MBP immunofluorescence signal intensity, or area covered across development and after three different manipulations altering cellular excitability or synaptic vesicle release in the murine brain. We also compared maturation of oligodendrocytes across development and after abrogation of synaptic vesicle release. Lastly, we analysed node of Ranvier length in the corpus callosum in all three conditions, and the g‐ratio in the spinal cord after LV SNAP25 cKO. As far as possible, we aimed to include measurements for all three manipulations, but the three manipulated cell populations (LV – Rbp4‐Cre, LVI – Ntsr1‐Cre and mixed LV/LVI after IUE) do not have identical axonal projections or location of cell bodies, so typically not all measurements were taken in the same regions across all brains. These multiple comparisons between strains and ages required considerable numbers of age‐matched and genotype‐matched animals. We aimed for at least *n *=* *3 brains per age/genotype, but this has not always been achieved. For ease of reference, Table S1 lists precisely the number of different animals/brains that contributed to each set of experiments.

Here we have presented evidence that the gross onset of first myelination in the murine brain does not appear to be affected by reduction in regulated vesicular release (SNAP25 cKO) in a small subset of cortical neurons. However, at P14, 1 week after the first signs of myelination in the brain, SNAP25 cKO in LVI neurons has a significant effect on the amount of MBP surrounding cKO axons, as measured by fluorescence intensity or area covered. This is accompanied by a significant reduction in the density of CC1^+^ or Olig2^+^CC1^+^ double‐positive, mature oligodendrocyte cells in M1 cortex; NoR length also tended to be reduced. The amount of myelination in the striatum of LV SNAP25 cKO brains was not altered, but there was a strong trend towards reduced NoR length in the callosum. There was no significant difference in oligodendrocyte precursor cell or mature oligodendrocyte density. Reduction in neuronal excitability via Kir2.1 expression had no marked effect on MBP intensity, but resulted in significantly reduced NoR length in the corpus callosum. IUE and control brains were not assessed for Olig2 and CC1 immunofluorescence, as all material had been used up in the other assessments and we did not want to conduct this quantification on a completely different set of brains.

The g‐ratio of SNAP25 cKO LV axons in the dorsal column of the spinal cord was significantly reduced, whereas mean axon diameter of measured axons was unaltered, suggesting more myelin wraps per myelinated axon. This is in contrast to the reduction in g‐ratio reported by Gibson and colleagues after channelrhodopsin‐based activity enhancement of cortical LV neurons (Gibson et al. 2014). We selected the time‐point of P18 for this assessment because immature myelin is less compacted than mature myelin, but we did not want to acquire the image too close to the age of P22, after which there is demonstrable degeneration of LV SNAP25 cKO axons in the spinal cord (Hoerder‐Suabedissen et al. 2018b).

The average g‐ratio was determined at the population level, as we were unable to find a method of fixation to preserve myelin ultrastructure while retaining tdTomato antigenicity for immuno‐gold labelling. Approximately 15% of LV NeuN^+^ neurons are labelled with *Rbp4‐Cre;Ai14*‐driven tdTomato (Hoerder‐Suabedissen et al. 2018b); however, we have no means of determining what proportion of spinal cord‐projecting LV neurons is tdTomato^+^. The dorsal column is densely packed with tdTomato^+^ axons (Hoerder‐Suabedissen et al. 2018b) and we assume that the dorsal column contains a mix of cKO and normal axons derived from Cre‐cells. We expect that the g‐ratio effect would be greater at the individual axon level if we could identify cKO and control axons. This is relevant, as Hines et al. (2015) demonstrated that nascent myelin sheath length is not affected if all axons are silenced by application of the voltage‐gated sodium channel blocker TTX, but is affected if only a subset of axons expresses the tetanus toxin light chain (TeNT), which cleaves synaptobrevin/VAMP and prevents vesicle exocytosis (Hines et al. 2015).

Unfortunately, we are unable to determine whether the LV SNAP25cKO axons are much more myelinated than normal axons or – more likely given the reduced myelination observed by Hines and colleagues after TeNT expression, and the increased myelin thickness observed around cortical LV axons after enhanced activity using channelrhodopsin by Gibson and colleagues – an excessive myelination of the remaining normal axons is the source of the effect observed here. As the g‐ratio histogram was shifted generally to the left (data not shown), rather than a new, big peak emerging for very small g‐ratios, we believe that the effect is more likely due to slightly more myelination in ‘bystander' non‐silenced axons, rather than excessive myelination in the cKO axons, which would be in keeping with the overall reduction in MBP signal observed in the brain after SNAP25 cKO. Mensch et al. (2015) also used TeNT expression in zebrafish spinal cord axons, and reported that overall fewer axons are myelinated at the EM level. We were unable to quantify the relative numbers of myelinated and unmyelinated axons in the present study due to the relatively lower resolution of scanning electron microscopes, making identification of unmyelinated axons unreliable. Thus, while we can demonstrate a population level effect of reduced g‐ratio in LV SNAP25 cKO spinal cords, we cannot demonstrate that this effect is specific to the cKO axons.

More clear cut is the effect of LVI SNAP25 cKO on myelination in the corpus callosum and in the cortex surrounding cKO cells. LVI SNAP25 cKO reduces the amount of myelin, which is in agreement with previous studies in zebrafish demonstrating reduced sheath length following expression of TeNT (Hines et al. 2015). Mensch et al. (2015) and Hines et al. (2015) both report that TeNT expression in zebrafish reduced the number of myelinated axons. Our measures of MBP signal intensity or area covered by it are unable to distinguish between these two possibilities of fewer axons being myelinated, or each axon being less myelinated. However, when measuring NoR length, in each image we could identify fewer NoR meeting our criteria in both the Kir2.1 IUE and LVI SNAP25 cKO brains compared with controls, despite similar levels of tdTomato^+^ axons in the callosum, which may suggest that fewer internodes are formed, or fewer axons are myelinated. However, it should be noted that the same SNAP25 cKO in LV axons did not result in reduced levels of MBP in the striatum, the only region that was sufficiently myelinated at P14, the time point of analysis, and contained LV axons.

In the present study, no significant effect on MBP levels was observed following Kir2.1 expression in a mixed LV/LVI cell population. This is in contrast to findings from both zebrafish and mouse. Hines et al. (2015) demonstrated in zebrafish that application of TTX reduces the number of myelinated axons. Mitew et al. (2018) demonstrated that expression of Kir2.1 in a callosally projecting cortical neuronal population reduced the number of axons that were surrounded by myelin rings in the callosum. Mitew and colleagues electroporated their Kir2.1 construct at E15.5, thus targeted a more superficial cortical neuronal population than we did in the present study. They also analysed their material at a slightly later stage (P20). Given the abundant differences between LV and LVI SNAP25 cKO, it is possible that the precise neuronal population targeted will make a significant difference to the effect observed.

In agreement with the absence of any differences in myelination, there were also no differences in oligodendrocyte density or maturational characteristics in LV SNAP25 cKO brains compared with controls. In LVI SNAP25 cKO brains, there were significantly fewer mature (CC1^+^ ) oligodendrocytes in M1 than in controls at P14, which ties in well with the reduction in myelination observed at the same age. However, the reduction in MBP immunofluorescence signal intensity was also observed in the P14 SNAP25 cKO striatum, although no significant differences in oligodendrocyte densities were observed. This could be the result of relative densities of affected fibres in the two regions; in the striatum, many unaffected fibres from other cortical populations also pass through, whereas in M1 the quantification was performed in the region of LVI cell bodies where their axonal processes are also at very high density. Our finding in LVI SNAP25 cKO brains at P14 suggests an overall delay in maturation (fewer CC1^+^ cells in cKO brains), which again is in contrast to Mitew et al. (2018), who found no differences in oligodendrocyte densities after Kir2.1 electroporation.

Node of Ranvier length has previously been proposed as a potential regulator of myelinated axon conduction speed, and modelling predicted that node length differences can alter conduction velocity by approximately 20% (Arancibia‐Carcamo et al. 2017). Additionally, it has been shown that altering the activity of axons can cause changes in nodal length (Huff et al. 2011; Trigo & Smith, 2015; Etxeberria et al. 2016). This suggests that NoR length could be modulated in activity‐dependent myelination plasticity; however, this has not been extensively investigated previously. The present study is, to our knowledge, the first to report node lengths of molecularly identified cortical neuronal populations (the LV and LVI control data). Additionally, we identified a strong trend towards reduced NoR length in the corpus callosum after either LV or LVI SNAP25 cKO and a significant reduction in NoR length after Kir2.1 IUE. Additionally, we could identify far fewer nodes after LVI SNAP25 cKO. A shorter node of Ranvier could be an indication of a compensatory mechanism of the ‘silenced' neurons, as a shorter NoR length could increase the velocity of the action potential (Arancibia‐Carcamo et al. 2017). Alternatively, it could indicate a deregulation of the myelin characteristics: too small a node will not be able to propagate the action potential, as there will not be sufficient voltage‐gated sodium channels for the action potential to gain enough strength at each node (Arancibia‐Carcamo et al. 2017). As the optimum node length for maximum velocity in these axon populations is currently unknown, it is not possible to make a distinction between the two mechanisms.

In the mouse optic nerve, Etxeberria et al. (2016) found both shortened internodes and a reduced conduction velocity associated with reduced neuronal activity due to monocular deprivation (Etxeberria et al. 2016). Here we were unable to measure internodal length, as we could not follow individual axons within the corpus callosum over such distances. The observed reduction in nodes in LVI SNAP25 cKO callosum compared with controls corroborates the reduction in MBP signal observed. This effect was not as pronounced after Kir2.1 IUE, where the reduction in MBP in the callosum was not significant.

Given the importance of myelination both in normal mammalian development and in disease conditions, an increased understanding of the mechanisms involved in this process is vital. Our work further supports the notion that myelination has two phases – intrinsic and then adaptive – in which active axons become more myelinated (Bechler et al. 2018). Most previous studies either have been *in vitro* or have involved *in vivo* manipulations in adult. This study utilised early onset, *in vivo,* manipulation of both regulated vesicular release and neuronal excitability, to study the effects of neuronal activity on subpopulations of cortical projections. We demonstrate differential effects in these models on overall MBP and on NoR length.

## Author contributions

Kim V. Korrell and Anna Hoerder‐Suabedissen contributed to experimental design, carried out experiments and conducted data analysis. Jolande Disser, Auguste Vadisiute and Kristina Parley carried out experiments, data analysis and contributed to manuscript writing. Mai‐Carmen Requena‐Komuro, Harriet Fodder and Charlotte Pollard cut tissue and carried out experiments. Graham Knott provided technical expertise and equipment and conducted the SEM imaging. Zoltán Molnár and Anna Hoerder‐Suabedissen initiated and planned the study, secured grant support and studentship and advised on data analysis and prepared the final manuscript.

## Supporting information


**Table S1**. The number of different animals used for each age and genotype for particular experiments.
