# Supplementary material for: Differential effect on myelination through abolition of activity‐dependent synaptic vesicle release or reduction of overall electrical activity of selected cortical projections in the mouse
Source: J Anat. 2019 Mar 22;235(3):452–67. doi: 10.1111/joa.12974 (PMC6704270; doi:10.1111/joa.12974)
Supplement: Supplementary file 1 — Table S1. The number of different animals used for each age and genotype for particular experiments. [file JOA-235-452-s001.docx]

|  | Kir2.1 IUE | | LVI SNAP25 cKO | | LV SNAP25 cKO | |
| --- | --- | --- | --- | --- | --- | --- |
|  | control | Kir2.1 | control | cKO | control | cKO |
| Onset of myelination | --- | --- | --- | --- | P6: n=2  P7: n=3  P8: n=4  P14: n=3 | P6: n=2  P8: n=2  P14: n=3 |
| Levels of MBP at P14 | cc: n=3  ctx: n=3 | cc: n=2  ctx: n=3 | cc: n=3  ctx: n=3  str: n=3 | cc: n=3  ctx: n=4  str: n=4 | str: n=3 | str: n=3 |
| Olig2 and CC1 IF | --- | --- | P8: n=2  P14: n=3  P21: n=3 | P8: n=3  P14: n=4  P21: n=4 | P8: n=4  P14: n=3  P21: n=7 | P8: n=2  P14: n=4  P21: n=7 |
| NoR length | n=6 | n=3 | n=4 | n=5 | n=3 | n=7 |
| g-ratio in spinal cord | --- | --- | --- | --- | n=5 | n=4 |

Table 1. This table indicates the number of different animals used for each age and genotype for particular experiments. Abbreviations: cc = corpus callosum, ctx = cerebral cortex, str = striatum, NoR = node of Ranvier, IF = immunofluorescence.
